# SAS profile correlations reveal SAS hierarchical nature and information content

**DOI:** 10.1371/journal.pone.0177309

**Published:** 2017-05-11

**Authors:** Yannick G. Spill, Michael Nilges

**Affiliations:** Structural Bioinformatics Unit, Department of Structural Biology and Chemistry, Institut Pasteur, 25 rue du Docteur Roux, 75015 Paris, France; Duke University, UNITED STATES

## Abstract

In structural biology, Small-Angle Scattering experiments (SAS) are unique, because although they provide low resolution data, they can be performed in closer-to-native conditions than those arising in X-Ray crystallography. A number of questions on SAS, however, remain unsolved, particularly in the light of modelling ensembles of conformers in solution. In this article, we study the ensemble average and covariance of SAS profiles analytically. Using this ensemble covariance, we demonstrate the hierarchical nature of SAS profiles. Furthermore, we show that the information content is not uniform and reaches its maximum in the intermediate *q* range. The arguments are generalized using microsecond-scale molecular dynamics trajectories of the lysozyme and on an ensemble of the intrinsically disordered protein p15PAF. We show that for highly flexible systems, the SAS profile is a representation of the ensemble of conformers in solution, and not that of one conformer in particular.

## Introduction

Biological small-angle scattering (SAS) of X-rays (SAXS) or neutrons (SANS) has regained interest, judging by the technical improvements made to beamlines recently [1, 2]. SAS experiments are easier to perform and in closer-to-native conditions than X-ray crystallography. Therefore, SAS is in a unique position for structural biologists, and the generalization of in-house SAXS experiments will only strengthen this position.

However convenient SAS experiments are, they only provide a limited amount of information. They are therefore often combined with other experiments to reach atomic resolution [3]. It is frequent to extract a number of parameters, such as the radius of gyration, the Porod exponent, or the volume of correlation [4–8]. Simple parameters, such as thos extracted from Kratky or Porod-Debye plots, can be used to assess the flexibility of a macromolecule [9]. Whether these or other parameters are independent of each other, and how they relate to the maximum number of independent points in a SAS profile [10–12] has not been studied in-depth.

It is also becoming clear that SAS measures conformational diversity [13]. More than 60% of all articles on the topic of SAXS ensembles have been published in the last five years, following the development of a number of methods for ensemble modelling (EOM [14], MES [15], BSS-SAXS [16], EROS [17], SES [18] and BE-SAXS [19]. In these methods, the SAS profile is almost always modelled as a weighted average of the profiles of the individual conformations. The different methods differ by the way they select the weights, and the number of conformations.

These methods are best suited to describe a small number of well-defined conformations present simultaneously in solution. However, cases where conformations vary continuously from one to the other are to be treated with much more care. As noted early on [14, 15], the obtained ensemble is then illustrative of the diversity of possible conformations. The number of conformations these methods propose are then not necessarily to be taken as granted, because the conformations are expected to have a strong internal variability [20]. In that respect, EROS [17] goes further in modelling continuous motion, because each conformation is already an average over a potentially large number of structures. Yet, the number of parameters, which in essence is three times the number of atoms times the number of structures, still becomes very large for such systems, and the risk of overfitting is not negligible. The most promising approach in that respect is the recently proposed BE-SAXS [19]. It proposes a generative model for the protein ensemble fitted on experimental SAXS data. This model therefore controls the expansion of the number of parameters. Yet it is unclear how that number of parameters can be extracted from it, and how to summarize the obtained distribution. Clearly, additional ways to represent continuous conformational variability would be welcome in the field. A first step is therefore to describe how structural variability affects SAS profiles in solution, which is the aim of this article.

## Materials and methods

**Fig 1:**
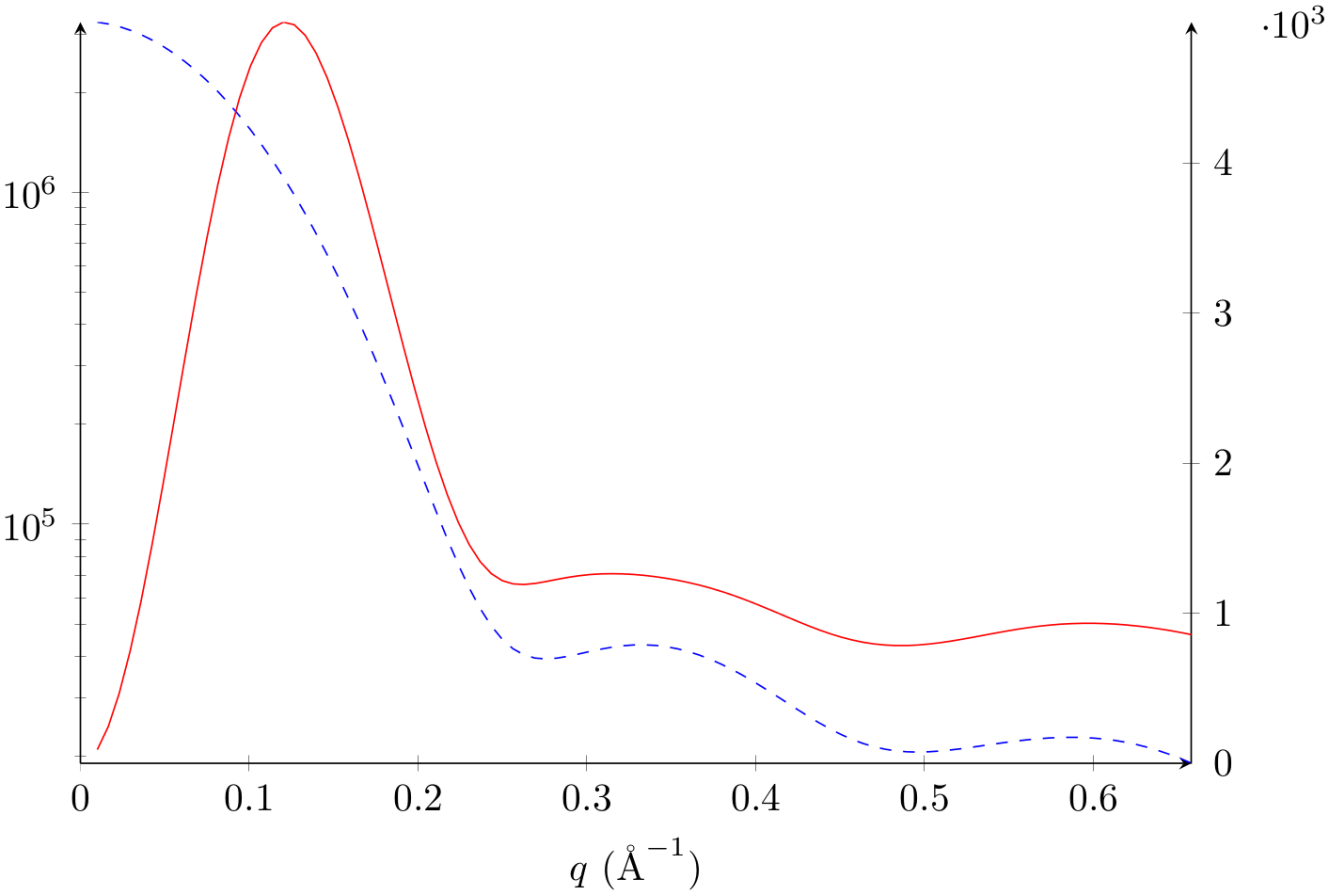
Lysozyme average SAS profile and standard deviation. Average SAS profile in blue dashed line, left axis, in arbitrary units, Eq 6. Standard deviation in solid red line, right axis, in arbitrary units, square root of Eq 11 with *q*_*i*_ = *q*_*j*_. All calculations use *τ* = 0.5 Å and FoXS form factors with *c*1 = 1 and *c*2 = 0 [23, 24].

**Fig 2:**
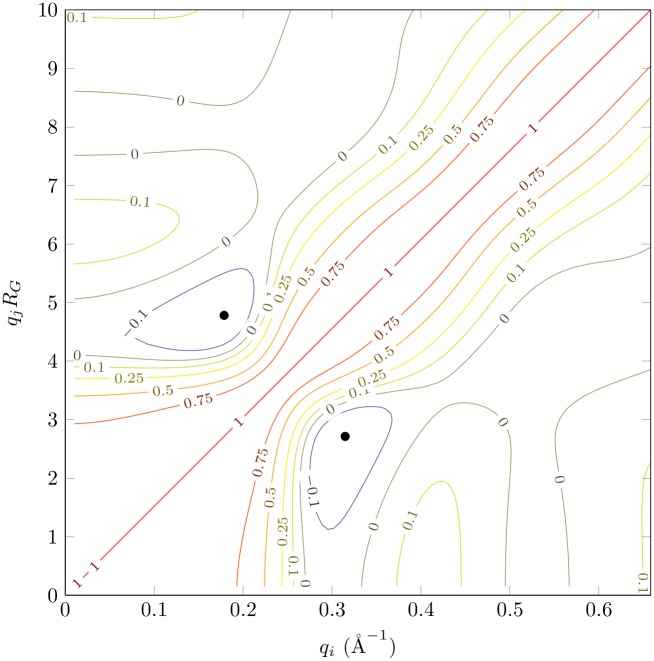
Contour plot of lysozyme SAS profile correlations. The correlations are given by *ρ*(*q*_*i*_, *q*_*j*_) (Eq 10). *R*_*G*_ = 15.2 Å. Smallest correlation is -0.28 and is indicated by a blue dot. Calculations use *τ* = 0.5 Å and FoXS form factors with *c*1 = 1 and *c*2 = 0 [23, 24].

We used the two 1 μs Molecular Dynamics (MD) simulations of the lysozyme described by Po-chia Chen and Jochen S. Hub [21], dropping the first 100 ns in each simulation. We performed the most likely alignment of the remaining frames using THESEUS [22]. This alignment produced a clash-free median structure for which the median atomic fluctuation was *τ* = 0.5 Å. It corresponds to the structure in the input which is closest to the center of the cluster. The median structure of the first simulation was taken as the center structure for all analytical calculations (and Figs 1 and 2).

Because we do not want to discuss the impact of solvation models on the calculations, we used a single model (FoXS [23, 24] with *c*_1_ = 1 and *c*_2_ = 0) to compute the SAS profiles of all structures of the simulations; more accurate solvation models should however be employed for practical applications when long MD trajectories are available [21, 25–27]. We refer to this as the correlated dataset. The numerical SAS variance of the lysozyme was then obtained by computing the variance matrix of these SAS profiles. We do not expect other solvation models to be very different from the two cases presented here.

Extension to intrinsically disordered proteins was performed on the p15PAF ensemble [28], available in the protein ensemble database under the accession code PED6AAA. We used the experimental profile of p15PAF, and the 4939 structures comprised in the ensemble. Individual SAS profiles were calculated with FoXS using *c*_1_ = 1 and *c*_2_ = 0.63.

## Results

### Ensemble average and covariance of SAS profiles

In this article, we relate the SAS profiles of conformers arising naturally in solution through thermal motion. We start with the Debye formula of the scattering intensity at momentum transfer *q* ≡ 4*π* sin *θ*/*λ* (scattering angle 2*θ* and wavelength *λ*) for an atomic structure comprising *N* atoms whose coordinates define the vector *X*

$$I_{X}\left(q\right)=\sum_{k=1}^{N}\sum_{l=1}^{N}f_{k}\left(q\right)f_{l}\left(q\right)\frac{\sin(qd_{kl})}{qd_{kl}}$$ (1)

where *d*_*kl*_ is the Euclidean distance between atoms *k* and *l* and *f*_*k*_(*q*) is the form factor of atom *k* at *q* [29]. Form factors used in this formula must include volume exclusion and solvent effects. Their definition is not a trivial task and falls outside of the scope of this article.

We now treat *X* as a random vector having 3*N* components. To model thermal motion we assume that *X* follows a Normal distribution around a mean structure *x*^∘^ with a diagonal covariance matrix such that atom *k* has variance $\tau_{k}^{2}$ along each of its coordinates. This simplifying assumption allows us to obtain analytical formulæ; it is the same as that used for the Debye-Waller temperature factors [30, 31]. We discuss generalizations thereof further down.

### Average intensity

The average intensity is computed by taking the mathematical expectation $E(I_{X}\left(q\right))$ of the intensity *I*_*X*_(*q*) over *X*. Using the linearity of the expectation in the Debye formula (Eq 1), we have

$$E\left(I_{X}\left(q\right)\right)=\sum_{k}f_{k}{\left(q\right)}^{2}+\sum_{k\neq l}f_{k}\left(q\right)f_{l}\left(q\right)E\left(\frac{\sin(qd_{kl})}{qd_{kl}}\right)$$ (2)

Therefore, we seek the average of $\frac{\sin(qd_{kl})}{qd_{kl}}$ for any pair of atoms *k* and *l*. It can be shown that in this case, the distance *d* between these two atoms follows a noncentral *χ* distribution with three degrees of freedom, whose probability density function is

$$p_{\chi }\left(d_{kl}|d_{kl}^{\circ },\tau_{k},\tau_{l}\right)=\frac{1}{\sqrt{2\pi (\tau_{k}^{2}+\tau_{l}^{2})}}\frac{d_{kl}}{d_{kl}^{\circ }}\left[\exp\left(-\frac{{\left(d_{kl}-d_{kl}^{\circ }\right)}^{2}}{2(\tau_{k}^{2}+\tau_{l}^{2})}\right)-\exp\left(-\frac{{\left(d_{kl}+d_{kl}^{\circ }\right)}^{2}}{2(\tau_{k}^{2}+\tau_{l}^{2})}\right)\right]$$ (3)

where $d_{kl}^{\circ }$ is the distance obtained when the atoms are at their average positions (see S1 Text). Without any approximation, we thus have

$$\forall q\geq 0,\;E\left(\frac{\sin(qd_{kl})}{qd_{kl}}\right)=\frac{\sin(qd_{kl}^{\circ })}{qd_{kl}^{\circ }}\exp\left(-q^{2}\frac{\tau_{k}^{2}+\tau_{l}^{2}}{2}\right)$$ (4)

This equality can then be inserted in the Debye equation to yield the SAS profile of the ensemble of conformations centered at structure *x*^∘^ described by the normal random variable *X*, now referred to as *thermal ensemble*.

$$E\left(I_{X}\left(q\right)\right)=\sum_{k=1}^{N}\sum_{l=1}^{N}\left(f_{k}\left(q\right)e^{-q^{2}\tau_{k}^{2}/2}\right)\left(f_{l}\left(q\right)e^{-q^{2}\tau_{l}^{2}/2}\right)\frac{\sin(qd_{kl}^{\circ })}{qd_{kl}^{\circ }}+\sum_{k=1}^{N}\left(1-e^{-q^{2}\tau_{k}^{2}}\right)f_{k}{\left(q\right)}^{2}$$ (5)

In the simple case where every atom has the same variance *τ*^2^, we have

$$E\left(I_{X}\left(q\right)\right)=e^{-q^{2}\tau^{2}}I_{x^{\circ }}\left(q\right)+\left(1-e^{-q^{2}\tau^{2}}\right)\sum_{k=1}^{N}f_{k}{\left(q\right)}^{2}$$ (6)

This result was obtained differently in 1932 by R. W. James [32], as recently rediscovered by P. B. Moore [33], who generalized it to anisotropic motion (*i*.*e*., arbitrary diagonal covariance matrix for *X*). It makes clear that the SAS profile of the thermal ensemble deviates from that of its center structure for momentum transfer values around and above 1/*τ*. For *τ* ≪ 1 Å, this effect is not within the measurable range of *q* values. However, in systems with large domain movements for which *τ* ≫ 1 Å, this effect becomes of prime importance. The fact that multiple different conformers coexist in solution can then be captured by SAS experiments. Indeed, the SAS curve of *x*^∘^ is then noticeably different from that of the thermal ensemble.

In addition, suppose that our system adopts two different conformations *A* and *B*, and that each of these is subject to thermal motions with deviations *τ*_*A*_ and *τ*_*B*_ such that *τ*_*A*_ ≪ *τ*_*B*_. This can happen, for example, when the system is made of two domains connected by a linker; *A* would be the state in which the two domains are in contact along a well-defined interaction surface, and *B* would be when the domains don’t interact. Then, assuming no interactions between *A* and *B* particles, the average intensity is a weighted sum of the intensities for *A* and *B*, each of them given by Eq 6. At low angle, the SAS profile contains information from both conformations. However, because the SAS intensities decay much faster for large *τ* values, the SAS profile of *A* will dominate that of *B* at high angle (assuming the populations of *A* and *B* are comparable). Therefore, in SAS, the higher *q* gets, the more we focus on well-defined conformations. There can be a number of them, but they must be well-defined. On the contrary, continuous conformational variability is more likely only to be noticed at low *q* values.

### Variance and correlation

In any case, because conformations of a thermal ensemble are related, there exist a number of rules that link their SAS profiles together. The SAS profile of one such conformation cannot deviate from Eq 5 in an arbitrary way. This is what we now show, by computing the covariance $V\left(I_{X}\left(q_{i}\right),I_{X}\left(q_{j}\right)\right)=E(I_{X}\left(q_{i}\right)I_{X}\left(q_{j}\right))-E(I_{X}\left(q_{i}\right))E(I_{X}\left(q_{j}\right))$ between the SAS profile at *q*_*i*_ and *q*_*j*_. For this purpose, we again use the Debye formula (Eq 1). The expectation of a product of intensities is

$$E\left(I_{X}\left(q_{i}\right)I_{X}\left(q_{j}\right)\right)=\sum_{kl}\sum_{mn}f_{k}\left(q_{i}\right)f_{l}\left(q_{i}\right)f_{m}\left(q_{j}\right)f_{n}\left(q_{j}\right)E\left(\frac{\sin(q_{i}d_{kl})}{q_{i}d_{kl}}\frac{\sin(q_{j}d_{mn})}{q_{j}d_{mn}}\right)$$ (7)

Then, we notice that

$$E\left(\frac{\sin(q_{i}d_{kl})}{q_{i}d_{kl}}\frac{\sin(q_{j}d_{mn})}{q_{j}d_{mn}}\right)=E\left(\frac{\sin(q_{i}d_{kl})}{q_{i}d_{kl}}\right)E\left(\frac{\sin(q_{j}d_{mn})}{q_{j}d_{mn}}\right)$$ (8)

when *k*,*l*,*m*,*n* describe four different atoms. Therefore, the terms that do not cancel out of the covariance calculation are 1) when *k* = *m* and *l* = *n*, *i*.*e*., the covariance of a distance with itself, which we call *autocovariance*; and 2) when *k* = *m* and *l* ≠ *n*, *i*.*e*. the covariance between two distances that share a common atom, which we call *cross-covariance*.

First, similar to the calculation of the average intensity, the autocovariance can be given in closed form. It however leads to a formula that is numerically unstable [34]. Second, the cross-covariance cannot be computed in closed form because the probability density function of the bivariate noncentral *χ* distribution is not known. Special cases exist for the bivariate noncentral *χ*^2^ probability density function [35] and the characteristic function [36], but the expectation still cannot be calculated.

We therefore seek an approximation to this distribution. A certain number of approaches exist [34, 37], but we use a more direct one (see S1 Text). It is based on a series expansion when all distances are much larger than *τ*. The bivariate noncentral *χ* distribution is then approximated as a bivariate normal distribution with mean vector **d**^**′**^ and covariance matrix **Σ**

$$d^{\prime }\equiv \left(\begin{array}{c} d_{kl}^{\circ }+\frac{\tau_{k}^{2}+\tau_{l}^{2}}{d_{kl}^{\circ }}\\ d_{kn}^{\circ }+\frac{\tau_{k}^{2}+\tau_{n}^{2}}{d_{kn}^{\circ }} \end{array}\right)\;\Sigma \equiv \left(\begin{array}{cc} \tau_{k}^{2}+\tau_{l}^{2} & \nu \tau_{k}^{2}\\ \nu \tau_{k}^{2} & \tau_{k}^{2}+\tau_{n}^{2} \end{array}\right)\;\nu \equiv \frac{d_{kl}^{\circ }\cdot d_{kn}^{\circ }}{d_{kl}^{\circ }d_{kn}^{\circ }}$$ (9)

Using this approximation, and to second order in *τ*/*d*^∘^, we can express the correlation and the covariance between the SAS profile at *q*_*i*_ and *q*_*j*_ (see S2 Text)

$$\rho (q_{i},q_{j})\equiv \frac{V(I_{X}\left(q_{i}\right),I_{X}\left(q_{j}\right))}{\sqrt{V\left(I_{X}\left(q_{i}\right),I_{X}\left(q_{i}\right)\right)V\left(I_{X}\left(q_{j}\right),I_{X}\left(q_{j}\right)\right)}}$$ (10)

$$V\left(I_{X}\left(q_{i}\right),I_{X}\left(q_{j}\right)\right)=V_{\text{auto}}\left(q_{i},q_{j}\right)+V_{\text{cross}}\left(q_{i},q_{j}\right)$$ (11)

$$V_{\text{auto}}\left(q_{i},q_{j}\right)\equiv \sum_{k}f_{k}\left(q_{i}\right)f_{k}\left(q_{j}\right)\sum_{l\neq k}f_{l}\left(q_{i}\right)f_{l}\left(q_{j}\right)V_{ij}^{\circ }\left(d_{kl}^{\circ }\right)$$ (12)

$$V_{\text{cross}}\left(q_{i},q_{j}\right)\equiv \sum_{k}f_{k}\left(q_{i}\right)f_{k}\left(q_{j}\right)\sum_{l\neq k}f_{l}\left(q_{i}\right)\sum_{n\neq k,l}f_{n}\left(q_{j}\right)V_{ij}\left(d_{\text{kl}}^{\circ },d_{\text{kn}}^{\circ }\right)$$ (13)

$$V_{ij}^{\circ }\left(d_{kl}^{\circ }\right)=\left(\tau_{k}^{2}+\tau_{l}^{2}\right)q_{i}q_{j}\sigma \left(q_{i}d_{kl}^{\circ }\right)\sigma \left(q_{j}d_{kl}^{\circ }\right)$$ (14)

$$V_{ij}\left(d_{\text{kl}}^{\circ },d_{\text{kn}}^{\circ }\right)=\nu \left(d_{\text{kl}}^{\circ },d_{\text{kn}}^{\circ }\right)\tau_{k}^{2}q_{i}q_{j}\sigma \left(q_{i}d_{kl}^{\circ }\right)\sigma \left(q_{j}d_{kn}^{\circ }\right)$$ (15)

$$\sigma \left(x\right)\equiv \frac{\text{d}}{\text{d}x}\frac{\sin(x)}{x}=\frac{1}{x}\left(\cos\left(x\right)-\frac{\sin(x)}{x}\right)$$ (16)

In all cases we studied, the standard deviation (SD) has the characteristic shape of Fig 1 (solid red line, see also S3 Text). The SD starts at zero, consistent with the fact that *I*(0) is proportional to the number of electrons, and is not impacted by conformational changes. It then quickly reaches a maximum, and then decreases to a plateau. On a relative scale therefore, the standard deviation represents a non-monotonically increasing proportion of the scattered intensity. This finding is consistent with those discussed for the average intensity (Eq 5), in that the conformational diversity is captured at wide angles. We do not expect different hydration models to produce significantly different standard deviations, unless they hydrate different conformers of the ensemble in a different way. However, in the most realistic cases, changes in conformation should cause the solvent shell to rearrange. The water density would therefore be impacted. Consequently, the standard deviation at *I*(0) could be be nonzero.

We now focus on the the correlation structure of the same SAS profile (Fig 2). In all cases we studied, correlations are strong close to the diagonal, and vanish when points are far apart. It is also frequent to observe at least one basin with negative correlations. The fact that points that are close together are highly correlated was expected. Indeed, this observation is a simple consequence of the predictable nature of SAS profiles on very short *q* scales.

Conversely, points that are far apart seem to be largely decorrelated. This fact demonstrates the hierarchical nature of SAS profiles [38]. Being a Fourier transform, the SAS profile describes the shape at low angle. At higher angle, it starts describing the quaternary structure and so forth. What these results suggest, is that SAS compartmentalizes these descriptions. Although individual atoms have a nonzero scattering contribution along the whole range of *q* values, collectively, a different trend emerges. For example, changes in the quaternary structure that do not modify the overall shape will not affect the onset of the SAS profile.

Another striking feature that can be seen in Fig 2 is that the bandwidth of this correlation matrix varies along the diagonal. Thus, neighboring points will be more or less correlated depending on their absolute position along the SAS profile. That is, the density of independent points along a SAS profile changes as *q* changes. In information theory, the mutual information of two random variables quantifies how much information one carries on the other. If we take two neighboring points along the SAS profile, their mutual information is

$$I\left(q_{i},q_{j}\right)=-\frac{1}{2}\log\left(1-\rho {\left(q_{i},q_{j}\right)}^{2}\right)$$ (17)

If the mutual information is high, *q*_*i*_ and *q*_*j*_ are strongly related, and consequently the information content of the SAS curve is lower in that region. But *ρ*(*q*_*i*_, *q*_*j*_) is directly related to the bandwidth of the correlation matrix. Therefore, the information content is not uniformly distributed along a SAS profile, and is larger when the bandwidth is smaller.

In all cases studied (see also S3 Text), the bandwidth is large at low *q*, becomes minimal between *qR*_*G*_ ∼ 3 − 6 and then broadens again at higher *q*, suggesting that the information content follows the opposite trends. This result confirms practical observations that the mid-*q* range (*qR*_*G*_ ∼ 3 − 6) is the most useful in structure refinement, while high-*q*, although beneficial, is not as valuable [39].

### Extension to correlated motion

The analytical model described until now makes the simplifying assumption that thermalization induces independent random normal displacements for each atom. Such an assumption has strong limitations [33, 40]. In particular, movements in solution are anisotropic, do not follow a normal distribution, and strong correlations between atoms or even protein domains can be expected. To a lesser extent, the bivariate noncentral chi distribution must be approximated to still obtain analytical results. This second-order approximation implies that the resulting covariance formulæare not exact but nonetheless very close, and in any case negligible compared to that of the anisotropic motion. In any case, more realistic representations of thermalization can be obtained with molecular dynamics (MD) simulations. We used the two 1 μs simulations of the lysozyme described by Po-chia Chen and Jochen S. Hub [21], from which we calculated the variance matrix.

**Fig 3:**
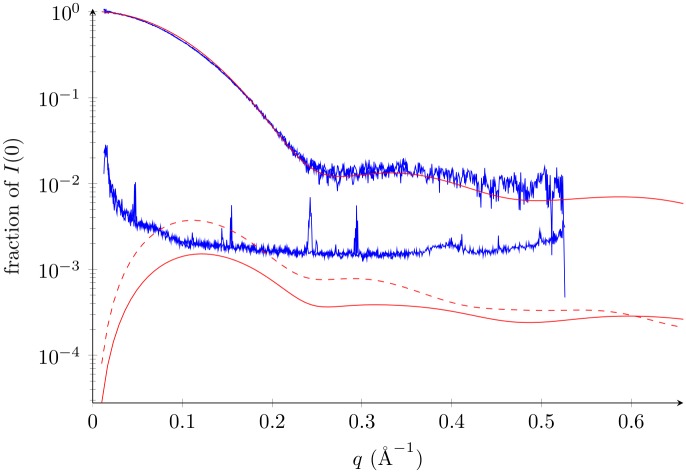
Lysozyme standard deviation compared to signal and noise. Standard deviation of correlated motion (*i*.*e*., first MD simulation) is dashed red line (see text for calculation). Standard deviation of independent motion (square root of Eq 11 with *τ* = 0.5 Å) is bottom solid red line. For reference, the experimental SAXS profile of the lysozyme (top) and its standard error (bottom) are shown in blue (bioisis code LYSOZP). Average SAS profile in the case of independent motion: top solid red.

Trends in the standard deviations are similar between correlated and independent motion (Fig 3). We see, however, that standard deviations are up to three times larger for correlated motion than for the independent case. They reach 4% of the SAS mean intensity on average, and can go up to 7% at *q* = 0.28 Å^−1^ in this example. These proportions are comparable with the experimental noise level, which commonly ranges from 0.1% to 10% in current experiments. This observation suggests that some structures, which arise naturally through thermal motion, can have a SAS profile that is noticeably different than that of their relatives.

They are, however, related through a set of rules which we now describe by looking at the correlations (see also S4 Text). Again, the correlated dataset is very comparable to the independent one. It has approximately the same location for the smallest correlation and the most narrow bandwidth. However, we can observe that 1) the smallest correlation is roughly twice as large, 2) the bandwidth is smaller overall, and 3) new correlation extrema appear between medium and high-*q*. We do not expect the just described features to change significantly between two hydration models. S4 Text shows the correlation matrix obtained from a second MD simulation. It is reasonable to expect that a change in hydration model would not cause larger differences than those observed between these two simulations.

The depicted correlations can be understood as forming a set of rules that must be satisfied by the SAS profile of any structure within the thermal ensemble. It comes to no surprise, therefore, that the region which has the highest coefficient of variation (*q* = 0.28 Å^−1^) is the one which is also the most constrained by the correlations. If in some conformers of the thermal ensemble, the SAS profile deviates by 7% from the ensemble SAS profile, then in doing so it must also deviate both at low and high *q* in a direction that is dictated by the covariances.

**Fig 4:**
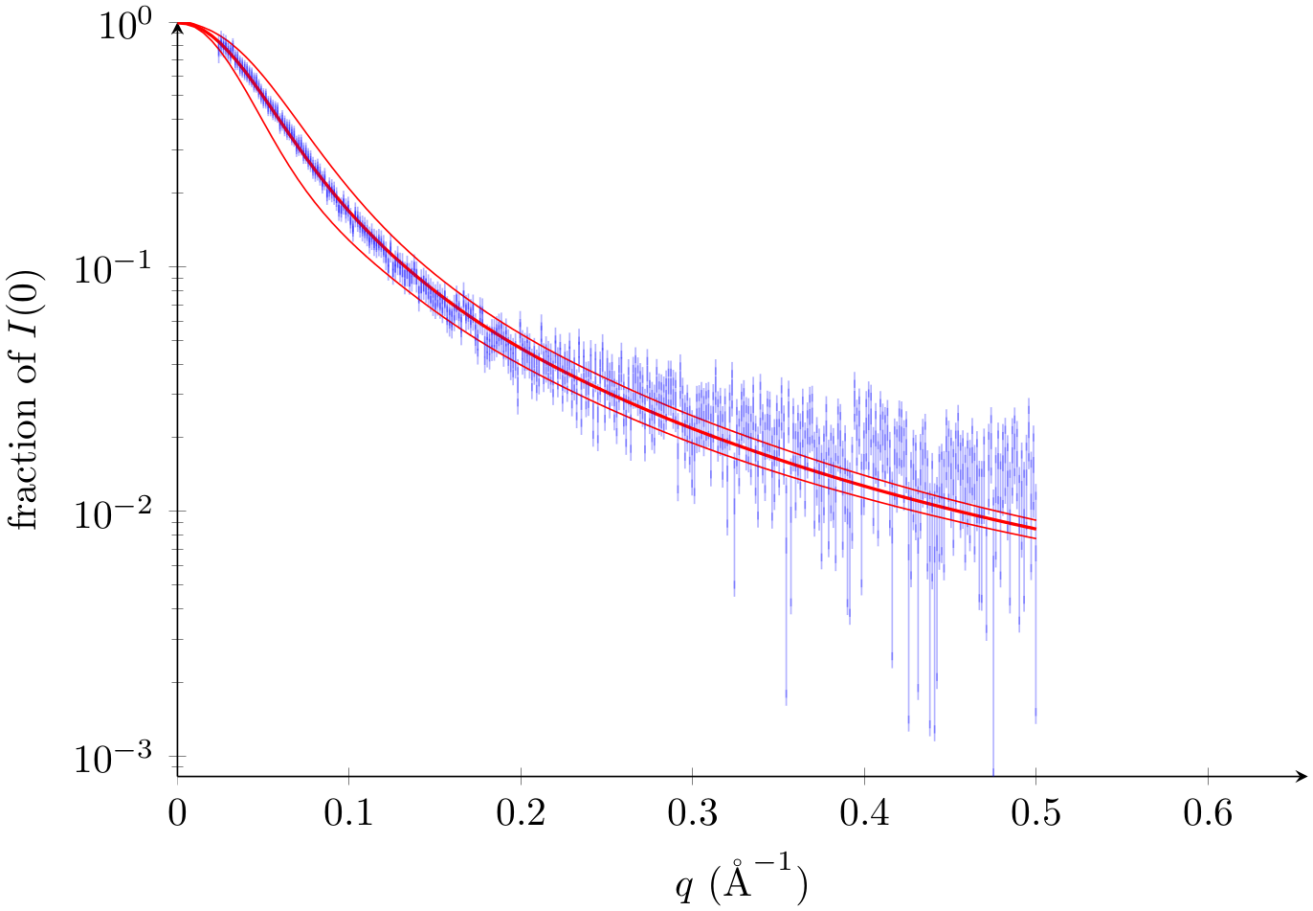
p15PAF experimental SAXS profile and ensemble average SAXS profile. p15PAF profile [28] (PED code PED6AAA) in blue. Ensemble average SAXS profile in thick red (Eq 6). 68% (1*σ*) confidence interval in red, ($\text{Eq }6\;\pm \;\sqrt{\text{Eq}\;11}$). The deposited ensemble contains 4939 structures. Individual SAXS profiles were calculated using FoXS with *c*1 = 1 and *c*2 = 0.63.

As can be seen, the variance grows with the square of the atomic motion (Eqs 14 and 15). For the lysozyme with correlated motion, these variances are comparable to experimental noise levels. For intrinsically disordered proteins in which atomic motion is an order of magnitude larger, this effect dominates the noise, as shown in the case of p15PAF (Fig 4, see also S5 Text) [28]. Therefore, the SAS profile of such a protein is not a static snapshot of one of its conformers, but instead captures its whole conformational complexity.

## Discussion

In this article, we describe the influence of continuous conformational changes on the SAS profile of a protein ensemble. To compute the quantities derived in this article, an atomic or pseudo-atomic model of the protein is needed. They describe how the SAS profile of a structure is modified if it is allowed to be flexible. The resulting SAS profile then contains information on the conformational diversity around that structure. It is however perfectly possible that this ensemble SAS profile be reproduced by a single, different structure. It is up to the modeling expert to determine whether it makes sense to include conformational flexiblity in the modeling or not. However, if the flexible ensemble and the other single structure both fit an experimental profile equally well, Occam’s razor would call for a description of the system by the simpler model. Therefore, and as already noted by others, ensemble modelling should only be performed if no satisfactory single conformation can be found.

In the second part of this article, the described SAS covariances are obtained through a long MD simulation. Care must be taken that this simulation is representative of the conformational diversity in solution. Multiple simulations should then yield the same covariance matrix. Unfortunately, even for very long simulations, such as the ones used here, small correlations are very difficult to converge. In our case, the second lysozyme simulation has the same overall covariance structure as described (trends in the variances, behaviour of the bandwidth, location of the global correlation minimum); but there are a number of differences as well: Standard deviations are up to four times larger than the independent case and reach up to 10% of the SAS mean intensity at *q* = 0.27 Å^−1^. Also, correlations between mid and high-*q* ranges do not stabilize (see supporting material). We suspect that these differences are mainly due to the fact that the second simulation has an enhanced loop motion [21].

We however hope that it will be possible to measure such a matrix experimentally, alleviating the need for an atomistic model of the protein. Through freezing of the particles in space with cryo-SAXS [41], it should be possible to measure SAS profiles of subsets of the thermal ensemble, and then infer the SAS covariance from them. This approach would work if the solution is sufficiently diluted so that the beam can interact with a small number of molecules, detecting fluctuations from thermodynamic averages. Through freezing in time with the X-ray free electron laser, the coherence of the beam might allow to reconstruct the SAS covariance directly, as already described three decades ago [42–44]. In essence, since for this experiment, the scattering pattern collected on the detector is not radially symmetric, correlations between and within annuli could be related to those of the SAS profile described in this article. We therefore hope that future developments will make the measure of SAS covariances possible.

## Conclusion

In this article, we have studied SAS profile correlations. We have shown they reveal the hierarchical nature of SAS profiles. We provided evidence that some portions of the experimental SAS profile are affected by ensemble averaging. Note that the SAS profile correlations described here have nothing in common with those estimated in a recent article, which are correlations of the noise of SAS experiments [45]. We, instead, estimate the correlations that are present within the signal itself.

First, a simple harmonic model of thermal motion allowed to obtain analytical expressions for the correlation between two points in a SAS profile. Second, the analysis of recently published microsecond MD simulations [21] allowed us to see that most trends in the correlations are conserved when thermal motion is modelled with more realism. Third, on the p15PAF structural ensemble [28], SAS profiles of different conformations within that ensemble differ more than the experimental error bar at *q*. Ensemble averaging can therefore be measured in that region. Last, we believe that these correlations could be measured experimentally with the help of cryo-SAXS or free-electron lasers.

Our developments show that SAS profiles are hierarchical, in the sense that successive regions of the SAS profile are decorrelated. Within these regions however, the knowledge of SAS correlations is essential to correctly describe highly flexible systems, such as intrinsically disordered proteins. We believe that in these systems, the SAS profile alone is not enough to grasp the system’s dynamics.

## Supporting information

S1 Text Normal approximation to noncentral *χ* distributions. Based on the derivations given in [34], this text describes the univariate and bivariate *χ* distributions, and their approximation when the variance is small.
(PDF)

S2 Text Calculation of the SAS variance. This text recapitulates and generalizes the derivation given in [34] to the case where each atom has its own variance.
(PDF)

S3 Text Three additional test cases. SAS mean intensities, standard deviations and correlations are reported for three additional protein test cases.
(PDF)

S4 Text Lysozyme MD simulations. Lysozyme correlations and standard deviation, as extracted from both MD simulations.
(PDF)

S5 Text p15PAF. Similar analysis on the ensemble of the p15PAF IDP.
(PDF)
