# Supplementary material for: SAS profile correlations reveal SAS hierarchical nature and information content
Source: PLoS One. 2017 May 11;12(5):e0177309. doi: 10.1371/journal.pone.0177309 (PMC5426712; doi:10.1371/journal.pone.0177309)
Supplement: S1 Text — Based on the derivations given in [34], this text describes the univariate and bivariate χ distributions, and their approximation when the variance is small. (PDF) [file pone.0177309.s001.pdf]

S1 Text: Normal approximation to noncentral χ distributions

Yannick G. Spill and Michael Nilges

1 Noncentral univariate χ

1.1 Derivation

Let $\mathbf{X}_k$ be the a random vector with three coordinates. Per construction, $\mathbf{X}_k \rightsquigarrow \mathcal{N}(\boldsymbol{\mu}_k, \mathbf{C}_k)$ where

$$\boldsymbol{\mu}_k \equiv \begin{pmatrix} x_k^\circ \\ y_k^\circ \\ z_k^\circ \end{pmatrix} \quad \mathbf{C}_k \equiv \begin{pmatrix} \tau_k^2 & 0 & 0 \\ 0 & \tau_k^2 & 0 \\ 0 & 0 & \tau_k^2 \end{pmatrix} \tag{1}$$

As a consequence, $\mathbf{d}_{kl} \equiv \mathbf{X}_l - \mathbf{X}_k$ follows a normal distribution with mean $\mathbf{d}_{kl}^\circ \equiv \boldsymbol{\mu}_l - \boldsymbol{\mu}_k$ and covariance matrix $\mathbf{C}_k + \mathbf{C}_l$. The Characteristic Function (CF) of the squared coordinates of $\mathbf{d}_{kl}$ can then be computed easily. Let c be one of the three coordinates of $\mathbf{d}_{kl}$, c° its mean and σ^2 its variance. Then

$$\mathbb{E}\left(e^{itc^2}\right) = \int_{\mathbb{R}} dc e^{itc^2} \frac{1}{\sqrt{2\pi}\sigma} e^{-\frac{1}{2\sigma^2}(c-c^\circ)^2} = \frac{1}{\sqrt{1-2it\sigma^2}} \exp\left(\frac{itc^{\circ 2}}{1-2it\sigma^2}\right) \tag{2}$$

We recognize the CF of a noncentral χ^2 distribution for the random variable c^2/σ^2 with one degree of freedom ($\nu = 1$) and noncentrality parameter $\lambda = c^{\circ 2}/\sigma^2$. The CF of a sum of random variables is their product, therefore $|d_{kl}|^2/(\tau_k^2 + \tau_l^2)$ follows a noncentral χ^2 distribution with $\nu = 3$ degrees of freedom and noncentrality parameter $\lambda = |d_{kl}^\circ|^2/(\tau_k^2 + \tau_l^2)$.

We just redemonstrated that the sum of the squares of three independent normal variables is a noncentral χ^2 distribution with three degrees of freedom. In this one-dimensional case, the Probability Density Function (PDF) is also known and can be obtained by an inverse fourier transform. After a change of variables, we finally have that $d_{kl} \equiv |\mathbf{d}_{kl}|$ has the following PDF

$$p_\chi(d_{kl}|d^\circ_{kl},\tau_{kl}) = \frac{1}{\sqrt{2\pi}\tau_{kl}}\frac{d_{kl}}{d^\circ_{kl}}\left[\exp\left(-\frac{(d_{kl}-d^\circ_{kl})^2}{2\tau_{kl}^2}\right) - \exp\left(-\frac{(d_{kl}+d^\circ_{kl})^2}{2\tau_{kl}^2}\right)\right] \tag{3}$$

where $d^\circ_{kl} \equiv \left|\mathbf{d}^\circ_{kl}\right|$ and $\tau^2_{kl} \equiv \tau^2_k + \tau^2_l$.

1.2 Approximation

We now seek a normal approximation to this distribution in the case where $\tau_{kl} \ll d^\circ_{kl}$. The mean and variance of the noncentral χ distribution is

$$\mathbb{E}(d_{kl}) = d^\circ_{kl} + \frac{\tau^2_{kl}}{d^\circ_{kl}} \qquad \mathbb{V}(d_{kl}) = \tau^2_{kl} \tag{4}$$

Therefore

$$p_\chi(d_{kl}|d^\circ_{kl},\tau_{kl}) \simeq p_{\mathcal{N}\left(d^\circ_{kl}+\frac{\tau^2_{kl}}{d^\circ_{kl}},\tau^2_{kl}\right)}(d_{kl}) \tag{5}$$

We can extend the integration domain to $\mathbb{R}$ since the probability mass on $\mathbb{R}_-$ of the normal distribution is negligible when $\tau_{kl} \ll d^\circ_{kl}$.

2 Noncentral bivariate χ

2.1 Attempted derivation

In an approach similar to section 1.1 (and with the same notations), we now seek the distribution of (d_{kl}, d_{kn}). $\mathbf{d_{kl}}$ and $\mathbf{d_{kn}}$ are correlated normal random variables. Let $\mathbf{G}$ be the 3×2 random normal matrix whose columns are $\mathbf{d_{kl}}$ and $\mathbf{d_{kn}}$, and let $\boldsymbol{\nu}_j{}^\top$ be the rows of $\mathbf{G}$. The $\boldsymbol{\nu}_j$ are normally distributed, and in particular, have a covariance matrix $\boldsymbol{\Sigma}_\star$ independent of j, shown below. Then, by definition,

$$\mathbf{V} \equiv \sum_{j=1}^{3} \boldsymbol{\nu}_j\boldsymbol{\nu}_j{}^\top = \begin{pmatrix} {d_{kl}}^2 & \mathbf{d_{kl}}\cdot\mathbf{d_{kn}} \\ \mathbf{d_{kl}}\cdot\mathbf{d_{kn}} & {d_{kn}}^2 \end{pmatrix} \tag{6}$$

has a noncentral Wishart distribution with three degrees of freedom, covariance matrix $\boldsymbol{\Sigma}_\star$ and noncentrality matrix $\boldsymbol{\Lambda}_\star$[1]

$$\boldsymbol{\Sigma}_\star \equiv \begin{pmatrix} \tau^2_{kl} & \tau^2_k \\ \tau^2_k & \tau^2_{kn} \end{pmatrix} \qquad \boldsymbol{\Lambda}_\star \equiv \begin{pmatrix} {d^\circ_{kl}}^2 & d^\circ_{kl}d^\circ_{kn}\cos\theta \\ d^\circ_{kl}d^\circ_{kn}\cos\theta & {d^\circ_{kn}}^2 \end{pmatrix} \tag{7}$$

where $\cos\theta$ is the cosine of the angle between $\mathbf{d}^\circ_{kl}$ and $\mathbf{d}^\circ_{kn}$. The PDF of the noncentral Wishart distribution has not been given explicitly in the general case. The case where

[1] Anderson and Girshick 1944; Anderson 1946; James 1955; Giri 2004, p. 231.

$\boldsymbol{\Lambda}$ is of rank 2 is called the planar case, and an expression of its PDF has been given as a central Wishart distribution multiplied by an infinite series of Bessel functions.[2] Its CF, however, has a simpler form, and can be obtained by computing the expectation of $e^{i \operatorname{tr}(\boldsymbol{W}\boldsymbol{V})}$ under the normal distribution of the V_j.[3] For ν degrees of freedom, covariance matrix $\boldsymbol{\Sigma}$ and noncentrality matrix $\boldsymbol{\Lambda}$, it is

$$\Psi_{\nu,\boldsymbol{\Sigma},\boldsymbol{\Lambda}}(\boldsymbol{W}) = \frac{\exp\left(\operatorname{tr}\left[i\boldsymbol{W}\left(\mathbb{1}_2 - 2i\boldsymbol{\Sigma}\boldsymbol{W}\right)^{-1}\boldsymbol{\Lambda}\right]\right)}{\det^{\nu/2}\left(\mathbb{1}_2 - 2i\boldsymbol{\Sigma}\boldsymbol{W}\right)} \tag{8}$$

The marginal of a distribution can be computed instantly with the CF, it is in fact sufficient to set the corresponding entries of the $\boldsymbol{W}$ matrix to zero. Therefore, the CF of the noncentral χ^2 distribution, which is the distribution of the diagonal elements of $\boldsymbol{V}$, is the same as that of the noncentral Wishart distribution provided we use a diagonal $\boldsymbol{W}$ matrix. We did not find it possible to express the CF or PDF of (d_{kl}, d_{kn}) from the CF of their squares. As we now show, this expression is however not necessary in our case.

2.2 Approximation

We seek an approximation PDF p to the noncentral bivariate χ distribution (PDF p_χ, which could not be expressed), that reduces to the univariate approximation by marginalization

$$\int p(d_{kl}, d_{kn}) \mathrm{d}d_{kl} = p_{\mathcal{N}\left(d^\circ_{kl} + \frac{\tau^2_{kl}}{d^\circ_{kl}}, \tau^2_{kl}\right)}(d_{kl}) \tag{9}$$

The following form

$$p(d_{kl}, d_{kn}) \equiv p_{\mathcal{N}(\mathbf{d}', \boldsymbol{\Sigma}_\rho)}(d_{kl}, d_{kn}) \qquad \mathbf{d}' \equiv \begin{pmatrix} d^\circ_{kl} + \frac{\tau^2_{kl}}{d^\circ_{kl}} \\ d^\circ_{kn} + \frac{\tau^2_{kn}}{d^\circ_{kn}} \end{pmatrix} \qquad \boldsymbol{\Sigma}_\rho \equiv \begin{pmatrix} \tau^2_{kl} & \rho\tau^2_k \\ \rho\tau^2_k & \tau^2_{kn} \end{pmatrix} \tag{10}$$

satisfies this constraint. We just need to find a suitable value for ρ. For that purpose, we compare the exact CF of (d^2_{kl}, d^2_{kn}) to their approximate one. The exact CF was derived in the previous section, and was shown to be equation 8 with $\nu = 3$, $\boldsymbol{\Sigma} = \boldsymbol{\Sigma}_\star$ and $\boldsymbol{\Lambda} = \boldsymbol{\Lambda}_\star$. Because the coefficient-wise square of a bivariate normal deviate is a noncentral bivariate χ^2 distribution with one degree of freedom, the CF of the approximation is equation 8 with $\nu = 1$, $\boldsymbol{\Sigma} = \boldsymbol{\Sigma}_\rho$ and $\boldsymbol{\Lambda} = \boldsymbol{\Lambda}_0 \equiv \mathbf{d}'\mathbf{d}'^\top$.

Similar to the previous section, we seek to match the moments of this approximation to those of the true distribution. The mean of both distributions are

[2] Anderson and Girshick 1944.
[3] Tourneret, Ferrari, and Letac 2005.

$$\mathbb{E}_p\left(\mathbf{d}\right) = \frac{1}{i}\Psi'_p(0) = \operatorname{diag}\left[\mathbf{\Lambda}_0 + \mathbf{\Sigma}_\rho\right] \qquad \mathbb{E}_{\chi^2}\left(\mathbf{d}\right) = \frac{1}{i}\Psi'_{\mathbf{\Lambda}_\star,\mathbf{\Sigma}_\star}(0) = \operatorname{diag}\left[\mathbf{\Lambda}_\star + 3\mathbf{\Sigma}_\star\right] \quad (11)$$

The difference of these moments does not depend on ρ and is zero to second order in τ/d°. Since ρ arises in the covariance matrix of the candidate distribution, it can be expected that the condition on ρ will come from equating the variances of both distributions. These variances are

$$\mathbb{V}_p\left(\mathbf{d}\right) = 2\mathbf{\Sigma}_\rho \circ \left(2\mathbf{\Lambda}_0 + \mathbf{\Sigma}_\rho\right) \qquad \mathbb{V}_{\chi^2}\left(\mathbf{d}\right) = 2\mathbf{\Sigma}_\star \circ \left(2\mathbf{\Lambda}_\star + 3\mathbf{\Sigma}_\star\right) \quad (12)$$

where $\circ$ is the coefficient-wise product. The diagonal terms of the difference of these matrices does not depend on ρ and is zero to second order in τ/d°, like previously. The outer-diagonal term of the difference is, to second order in τ/d°, equal to

$$2\tau_{kl}^2 d_{kl}^\circ d_{kn}^\circ (\cos\theta - \rho) \quad (13)$$

This term, equated to zero, leads to the unique solution

$$\rho = \cos\theta \quad (14)$$

In conclusion, the probability density function

$$p(d_{kl}, d_{kn}) \equiv p_{\mathcal{N}(\mathbf{d}',\mathbf{\Sigma}_\rho)}(d_{kl}, d_{kn}) \quad (15)$$

$$\mathbf{d}' \equiv \begin{pmatrix} d_{kl}^\circ + \frac{\tau_{kl}^2}{d_{kl}^\circ} \\ d_{kn}^\circ + \frac{\tau_{kn}^2}{d_{kn}^\circ} \end{pmatrix} \qquad \mathbf{\Sigma}_\rho \equiv \begin{pmatrix} \tau_{kl}^2 & \rho\tau_k^2 \\ \rho\tau_k^2 & \tau_{kn}^2 \end{pmatrix} \qquad \rho = \cos\theta \quad (16)$$

approximates that of the noncentral bivariate χ distribution with three degrees of freedom for large distances with respect to τ. This approximation matches the first two moments of the true distribution to second order in τ/d°.

References

Anderson, T. W. (1946). “The Non-Central Wishart Distribution and Certain Problems of Multivariate Statistics”. English. In: *Ann. Math. Stat.* 17.4, pp. 409–431 (cit. on p. 2).

Anderson, T. W. and M. A. Girshick (1944). “Some Extensions of the Wishart Distribution”. English. In: *Ann. Math. Stat.* 15.4, pp. 345–357 (cit. on pp. 2, 3).

Giri, Narayan C. (2004). *Multivariate statistical analysis*. Vol. 171. CRC Press (cit. on p. 2).

James, A. T. (1955). “The Non-Central Wishart Distribution”. In: *Proceedings of the Royal Society of London*. Vol. 229. Series A, Mathematical and Physical Sciences. The Royal Society, pp. 364–366 (cit. on p. 2).

Tourneret, J.-Y., A. Ferrari, and G. Letac (2005). “The noncentral wishart distribution: properties and application to speckle imaging”. In: *IEEE/SP 13th Workshop on Statistical Signal Processing*, pp. 924–929. DOI: 10.1109/SSP.2005.1628726 (cit. on p. 3).
