# Supplementary material for: SAS profile correlations reveal SAS hierarchical nature and information content
Source: PLoS One. 2017 May 11;12(5):e0177309. doi: 10.1371/journal.pone.0177309 (PMC5426712; doi:10.1371/journal.pone.0177309)
Supplement: S2 Text — This text recapitulates and generalizes the derivation given in [34] to the case where each atom has its own variance. (PDF) [file pone.0177309.s002.pdf]

S2 Text: Calculation of the SAS variance

Yannick G. Spill and Michael Nilges

1 Autocovariance

We start with the calculation of the autocovariance

$$V_{ij}(d^\circ) \equiv \mathbb{V}\left(\frac{\sin(q_i d)}{q_i d}, \frac{\sin(q_j d)}{q_j d}\right) \tag{1}$$

Define

$$g(d) \equiv \frac{\sin(q_1 d)}{q_1 d} \quad h(d) \equiv \frac{\sin(q_2 d)}{q_2 d} \quad f(d) \equiv g(d)h(d) \tag{2}$$

Then, to second order around $d' \equiv d^\circ + \frac{\tau^2}{d^\circ}$

$$f(d) \simeq f + f'(d - d') + f''\frac{(d - d')^2}{2} \tag{3}$$

where functions without arguments are understood as being evaluated at d', *i.e.*, f stands for $f(d')$ *etc*. For $d \equiv d_{kl}$, τ is understood as τ_{kl}. Therefore

$$\mathbb{E}\left(f(d)\right) \equiv \int_0^{+\infty} f(d) p_\chi(d|d^\circ, \tau) dd = f + f' T_1 + f'' T_2 \tag{4}$$

where the coefficients T_1 and T_2 are

$$T_1 \equiv \mathbb{E}(d - d') = 0 \tag{5}$$

$$T_2 \equiv \mathbb{E}\left(\frac{(d - d')^2}{2}\right) = \frac{\tau^2}{2} \tag{6}$$

The expressions for g and h are similar with the same T_i coefficients. Note that these coefficients have the same value with the exact distribution or with its approximation since we matched the first two moments. The previous equation thus reduces to

$$\mathbb{E}\left(f(d)\right) = f + f'' T_2 \tag{7}$$

We now express the derivatives of f as a function of those of g and h

$$f = gh \tag{8}$$
$$f' = g'h + gh' \tag{9}$$
$$f'' = g''h + gh'' + 2g'h' \tag{10}$$

Now to second order

$$\mathbb{E}\left(g(d)\right)\mathbb{E}\left(h(d)\right) = gh + (g''h + gh'')T_2 \tag{11}$$

Finally, the autocovariance is

$$V_{ij}(d^\circ) = \tau^2 q_i q_j \sigma(q_i d^\circ)\sigma(q_j d^\circ) \tag{12}$$

with σ the derivative of sinc.

2 Cross-covariance

Now, we derive the cross-covariance

$$V_{ij}(\mathbf{d}^\circ_{kl}, \mathbf{d}^\circ_{kn}) \equiv \mathbb{V}\left(\frac{\sin(q_i d_{kl})}{q_i d_{kl}}, \frac{\sin(q_j d_{kn})}{q_j d_{kn}}\right) \tag{13}$$

Similarly to the autocovariance, define

$$g(d) \equiv \frac{\sin(q_1 d)}{q_1 d} \quad h(d) \equiv \frac{\sin(q_2 d)}{q_2 d} \quad f(\mathbf{d}) \equiv g(d_{kl})h(d_{kn}) \tag{14}$$

Then, to second order around $\mathbf{d}'$

$$f(\mathbf{d}) \simeq f + \nabla^\top f\,(\mathbf{d} - \mathbf{d}') + \frac{1}{2}(\mathbf{d} - \mathbf{d}')^\top \Delta f\,(\mathbf{d} - \mathbf{d}') \tag{15}$$

where functions without arguments are understood as being evaluated at $\mathbf{d}'$, *i.e.*, f stands for $f(\mathbf{d}')$ *etc*. Therefore

$$\mathbb{E}\left(f(\mathbf{d})\right) \equiv \int_0^{+\infty} f(\mathbf{d}) p_\chi(\mathbf{d}|\mathbf{d}^\circ, \tau) d\mathbf{d} = f + \nabla^\top f\,\mathbf{R}_1 + \mathrm{tr}\left(\boldsymbol{\Delta} f\,\mathbf{R}_2\right) \tag{16}$$

where the coefficients $\mathbf{R}_1$ and $\mathbf{R}_2$ are computed using the approximated distribution

$$\mathbf{R}_1 \equiv \mathbb{E}(\mathbf{d} - \mathbf{d}') = 0 \tag{17}$$
$$\mathbf{R}_2 \equiv \frac{1}{2}\mathbb{E}\left((\mathbf{d} - \mathbf{d}')(\mathbf{d} - \mathbf{d}')^\top\right) = \frac{1}{2}\boldsymbol{\Sigma}_\rho \tag{18}$$

with $\rho = \cos\theta$. The expressions for g and h are similar

$$\mathbb{E}\left(g(d_{kl})\right) = g + g''\frac{\tau_{kl}^2}{2} \tag{19}$$

$$\mathbb{E}\left(h(d_{kn})\right) = h + h''\frac{\tau_{kn}^2}{2} \tag{20}$$

with g understood as $g(d_{kl}^\circ)$ and h as $h(d_{kn}^\circ)$ respectively. We express the derivatives of f as a function of those of g and h

$$f = gh \quad \nabla f = \begin{pmatrix} g'h \\ gh' \end{pmatrix} \quad \boldsymbol{\Delta} f = \begin{pmatrix} g''h & g'h' \\ g'h' & gh'' \end{pmatrix} \tag{21}$$

Now to second order, we have

$$\mathbb{E}\left(g(d)\right)\mathbb{E}\left(h(d)\right) = f + g''h\frac{\tau_{kl}^2}{2} + gh''\frac{\tau_{kn}^2}{2} \tag{22}$$

Finally, the cross-covariance is

$$V_{ij}(\mathbf{d}_{kl}^\circ, \mathbf{d}_{kn}^\circ) = \rho\tau_k^2 q_i q_j \sigma(q_i d_{kl}^\circ)\sigma(q_j d_{kn}^\circ) \tag{23}$$

with σ the derivative of sinc and $\rho = \frac{\mathbf{d}_{kl}^\circ \cdot \mathbf{d}_{kn}^\circ}{d_{kl}^\circ d_{kn}^\circ}$.
