# Supplementary material for: SAS profile correlations reveal SAS hierarchical nature and information content
Source: PLoS One. 2017 May 11;12(5):e0177309. doi: 10.1371/journal.pone.0177309 (PMC5426712; doi:10.1371/journal.pone.0177309)
Supplement: S3 Text — SAS mean intensities, standard deviations and correlations are reported for three additional protein test cases. (PDF) [file pone.0177309.s003.pdf]

S3 Text: Three additional test cases

Yannick G. Spill and Michael Nilges

Here, we report three additional test cases extracted from a recent SAXS benchmark[1]

- ATG7: Ubiquitin-like modifier-activating enzyme, ATG7 C-terminal domain[2]
- PYR1P: Abscisic acid receptor PYR1[3]
- SOD: Superoxide dismutase[4]

References

Nishimura, Noriyuki, Kenichi Hitomi, Andrew S. Arvai, Robert P. Rambo, Chiharu Hitomi, Sean R. Cutler, Julian I. Schroeder, and Elizabeth D. Getzoff (2009). "Structural Mechanism of Abscisic Acid Binding and Signaling by Dimeric PYR1". In: *Science* 326.5958, pp. 1373–1379. DOI: 10.1126/science.1181829 (cit. on p. 1).

Schneidman-Duhovny, Dina, Michal Hammel, John A. Tainer, and Andrej Sali (2013). "Accurate SAXS Profile Computation and its Assessment by Contrast Variation Experiments". In: *Biophys. J.* 105.4, pp. 962–974. DOI: 10.1016/j.bpj.2013.07.020 (cit. on p. 1).

Shin, David S., Michael DiDonato, David P. Barondeau, Greg L. Hura, Chiharu Hitomi, J. Andrew Berglund, Elizabeth D. Getzoff, S. Craig Cary, and John A. Tainer (2009). "Superoxide Dismutase from the Eukaryotic Thermophile Alvinella pompejana: Structures, Stability, Mechanism, and Insights into Amyotrophic Lateral Sclerosis". In: *J. Mol. Biol.* 385.5, pp. 1534–1555. DOI: 10.1016/j.jmb.2008.11.031 (cit. on p. 1).

Taherbhoy, Asad M., Stephen W. Tait, Stephen E. Kaiser, Allison H. Williams, Alan Deng, Amanda Nourse, Michal Hammel, Igor Kurinov, Charles O. Rock, Douglas R. Green, and Brenda A. Schulman (2011). "Atg8 Transfer from Atg7 to Atg3: A Distinctive E1-E2 Architecture and Mechanism in the Autophagy Pathway". In: *Mol. Cell* 44.3, pp. 451–461. DOI: 10.1016/j.molcel.2011.08.034 (cit. on p. 1).

---

[1] Schneidman-Duhovny et al. 2013.
[2] Taherbhoy et al. 2011.
[3] Nishimura et al. 2009.
[4] Shin et al. 2009.

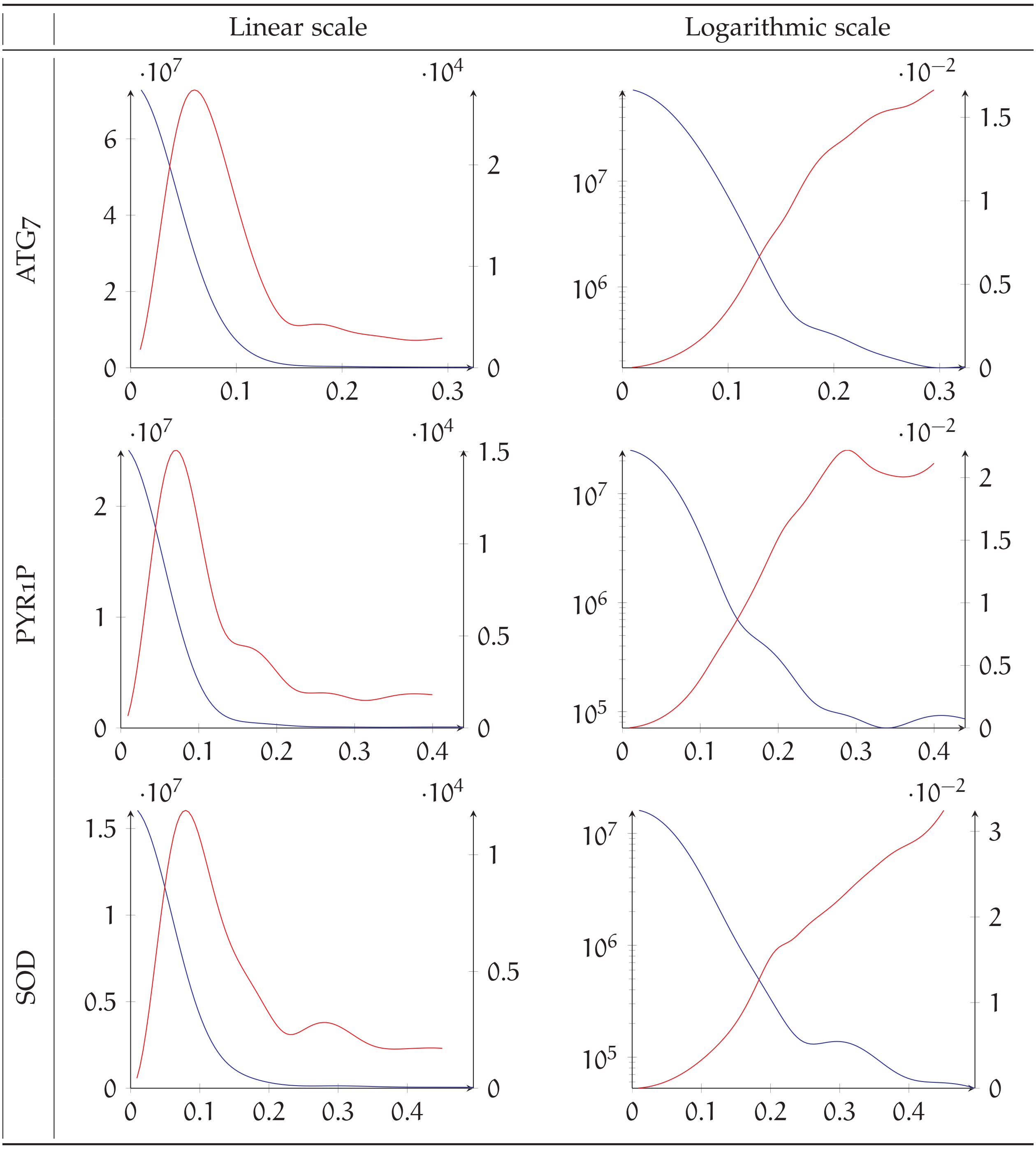

Figure 1: Mean (blue) and standard deviation (red) of the SAXS profile of three different protein systems. X-axis is $q(\text{Å}^{-1})$. Left y-axis is SAXS intensity (A.U.), right y-axis is standard deviation (left panel, A.U.) or coefficient of variation $V \equiv \sigma/\mu$ (right panel).

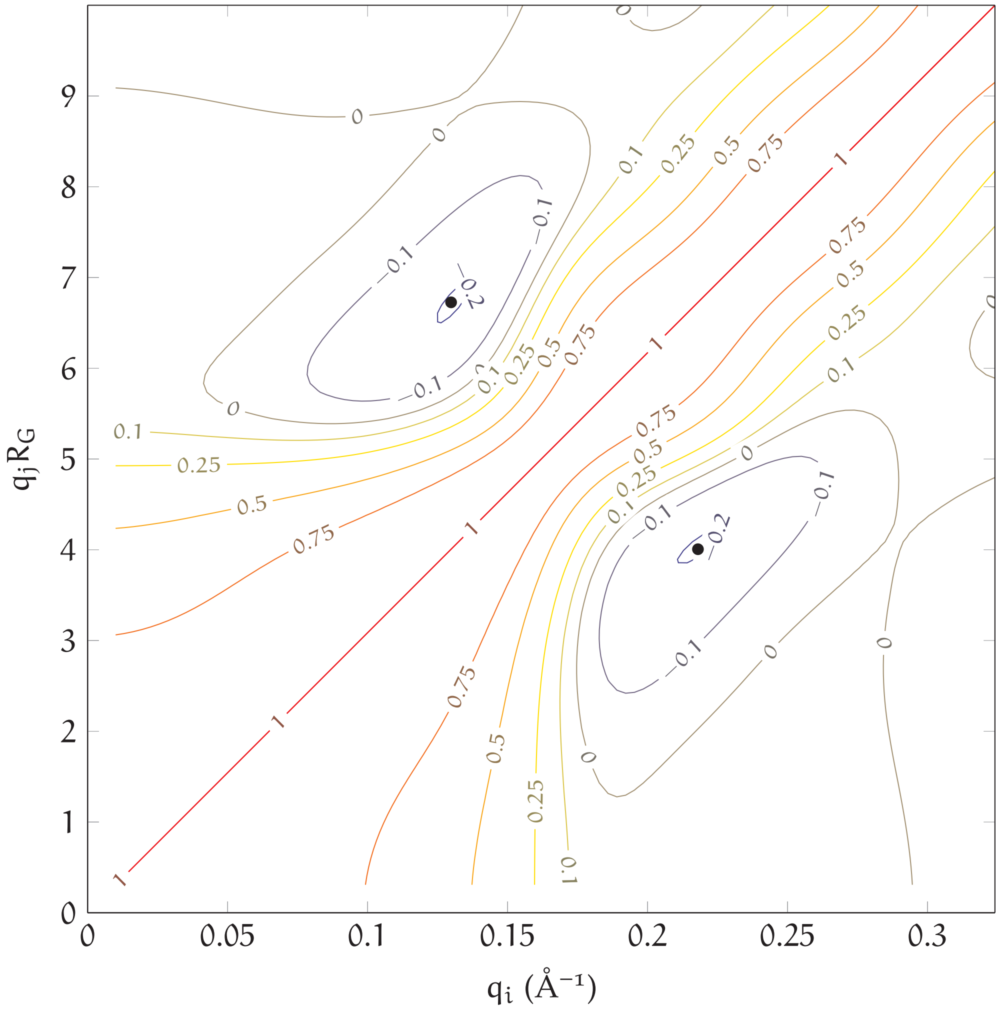

Figure 2: ATG7

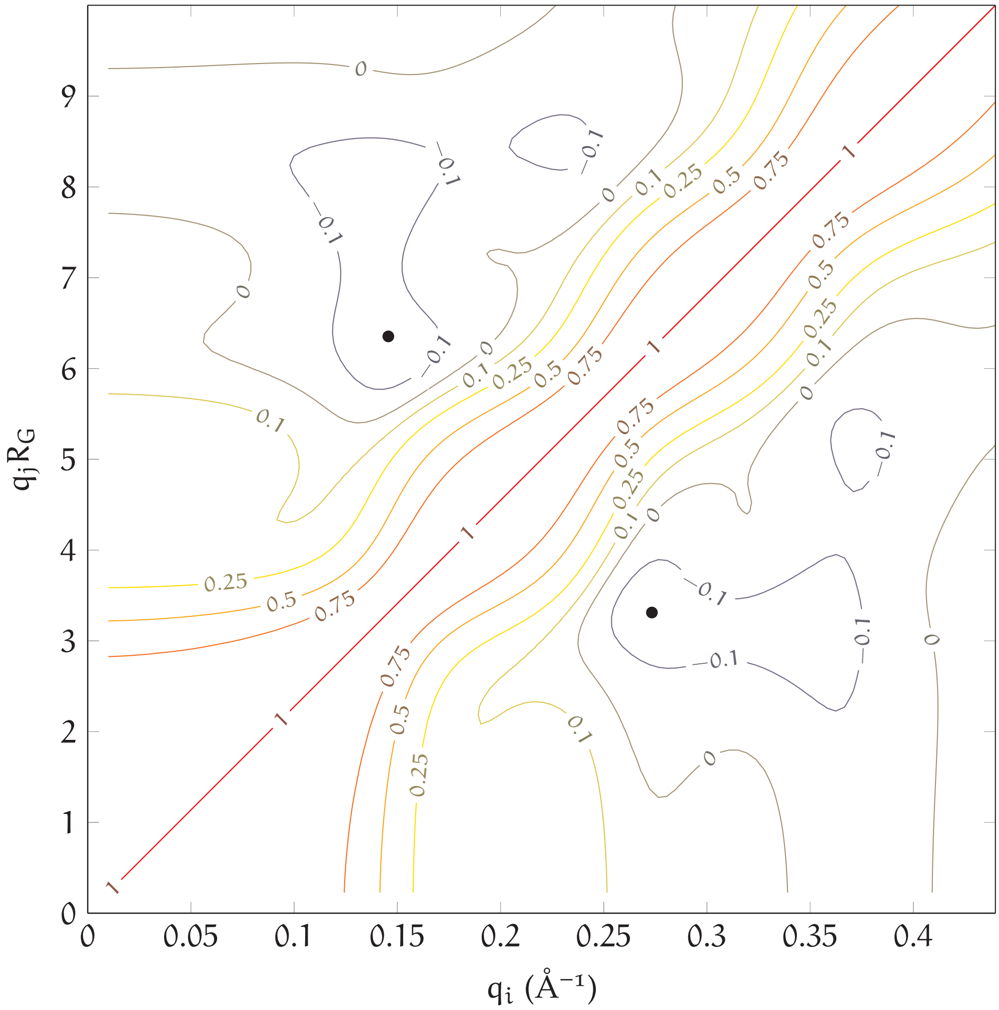

Figure 3: PYR1P

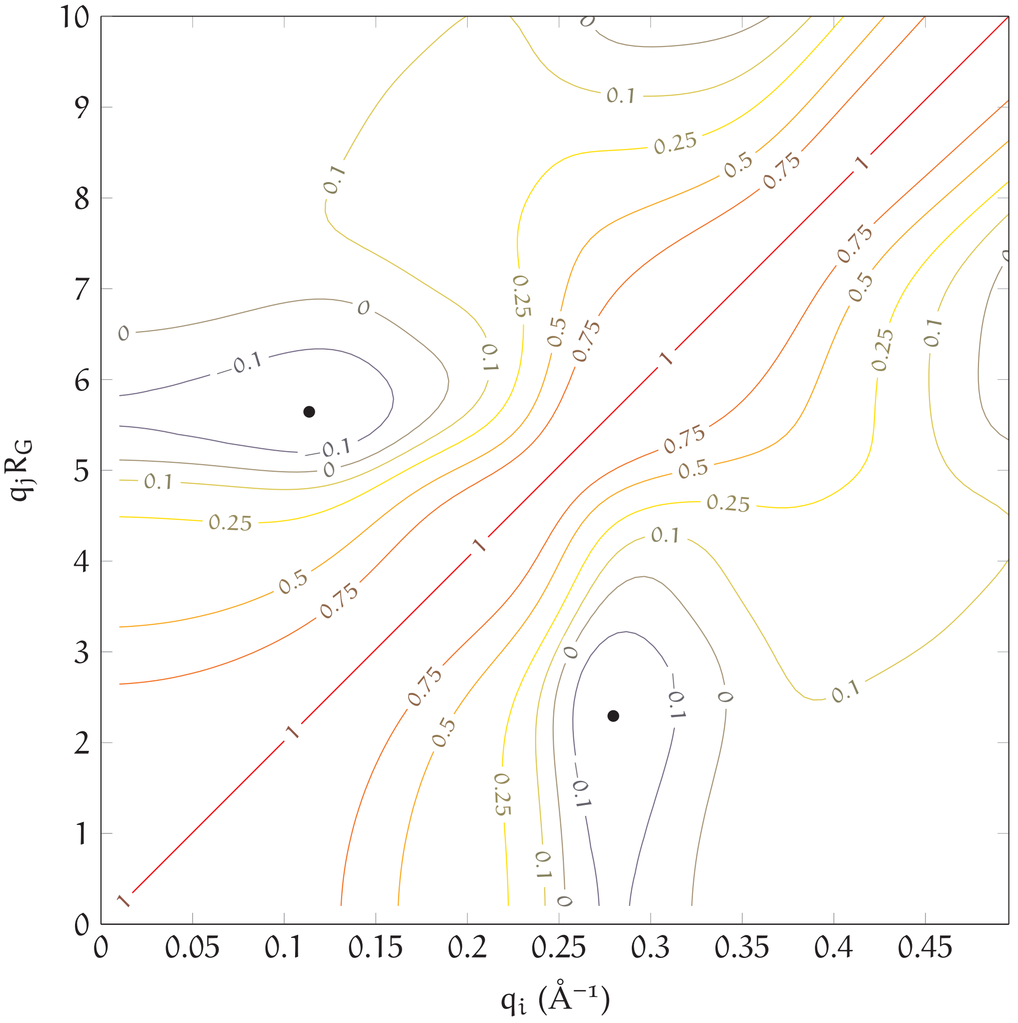

Figure 4: sod
