# Supplementary material for: SAS profile correlations reveal SAS hierarchical nature and information content
Source: PLoS One. 2017 May 11;12(5):e0177309. doi: 10.1371/journal.pone.0177309 (PMC5426712; doi:10.1371/journal.pone.0177309)
Supplement: S4 Text — Lysozyme correlations and standard deviation, as extracted from both MD simulations. (PDF) [file pone.0177309.s004.pdf]

S4 Text: Lysozyme MD simulations

Yannick G. Spill and Michael Nilges

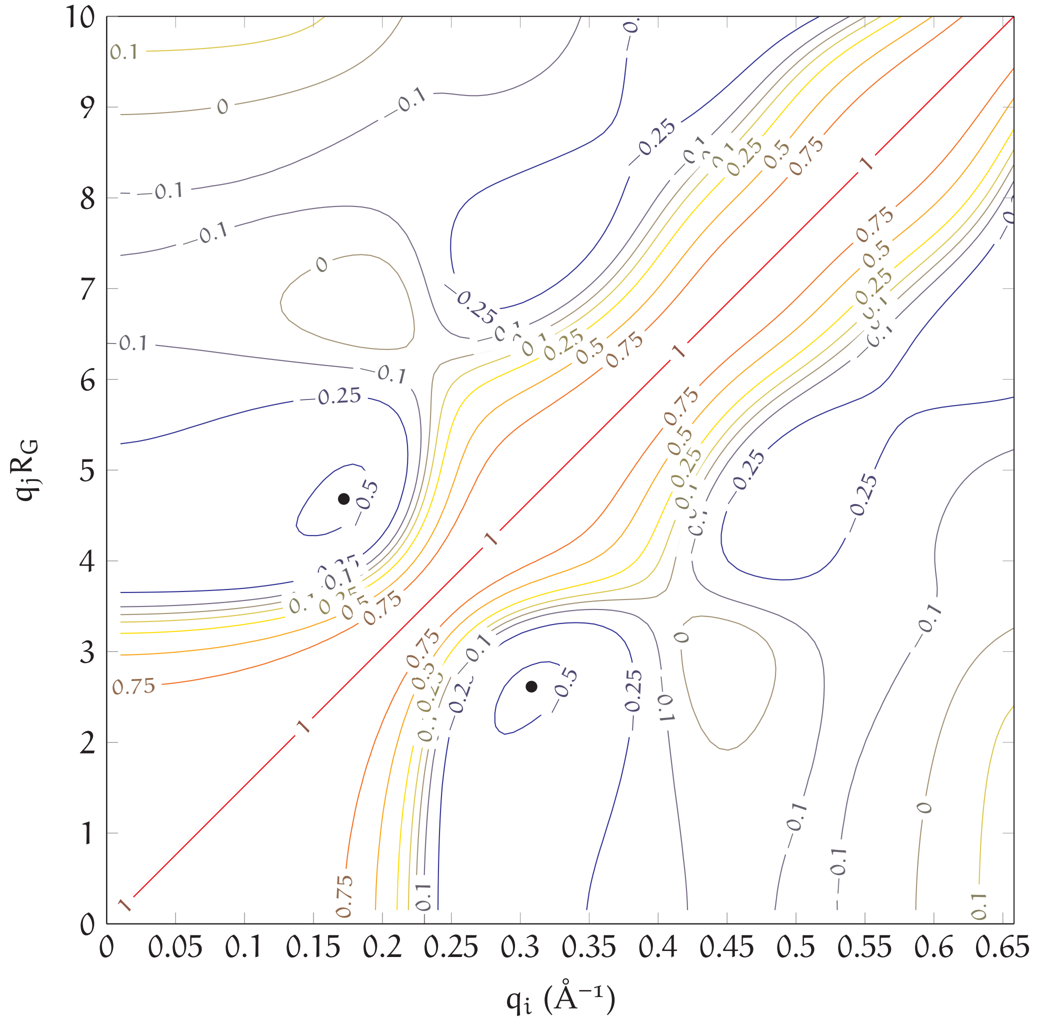

Figure 1: Lysozyme SAS profile numerical correlations using MD (see text). $R_G = 15.2$ Å. Smallest correlation is −0.53 and is indicated by a blue dot.

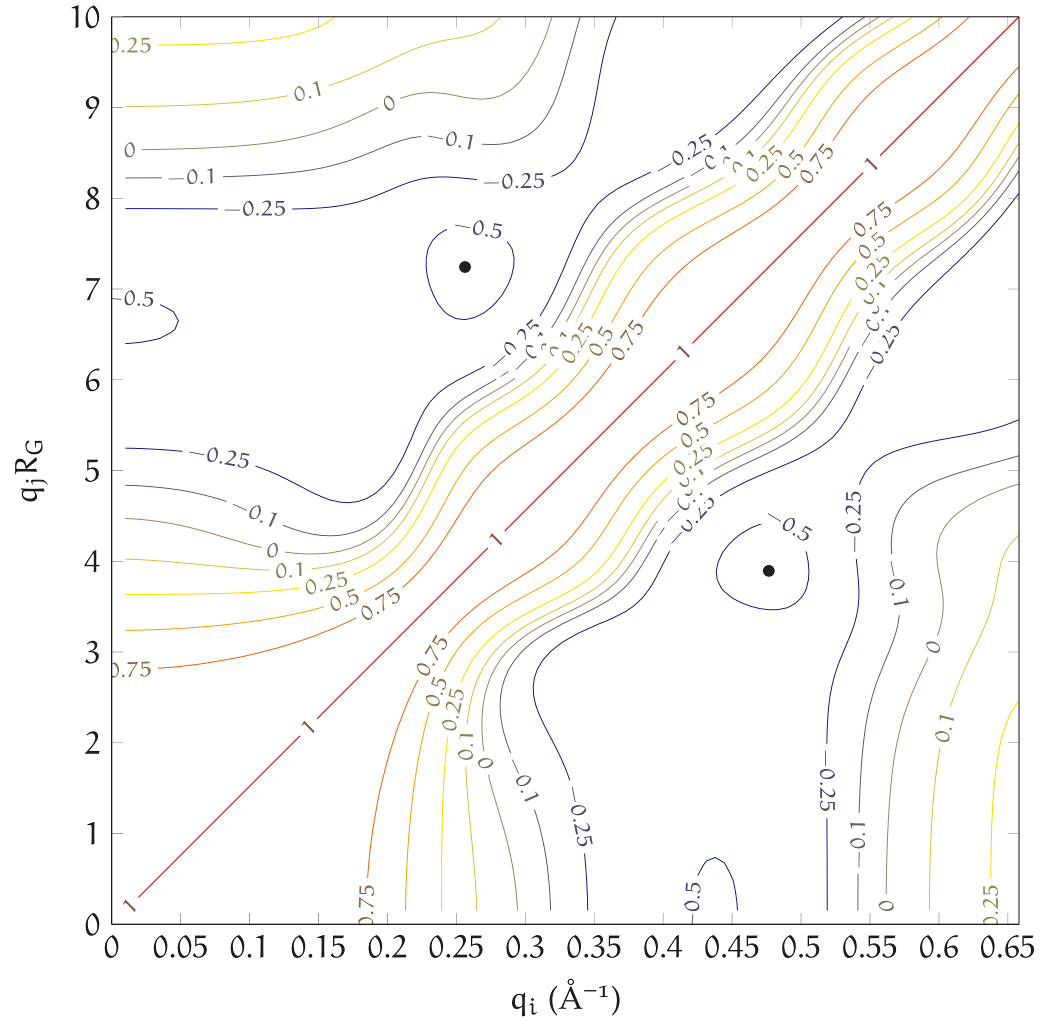

Figure 2: Lysozyme SAS profile numerical correlations using second lysozyme simulation. $R_G = 15.2$ Å. Smallest correlation is −0.58 and is indicated by a blue dot.

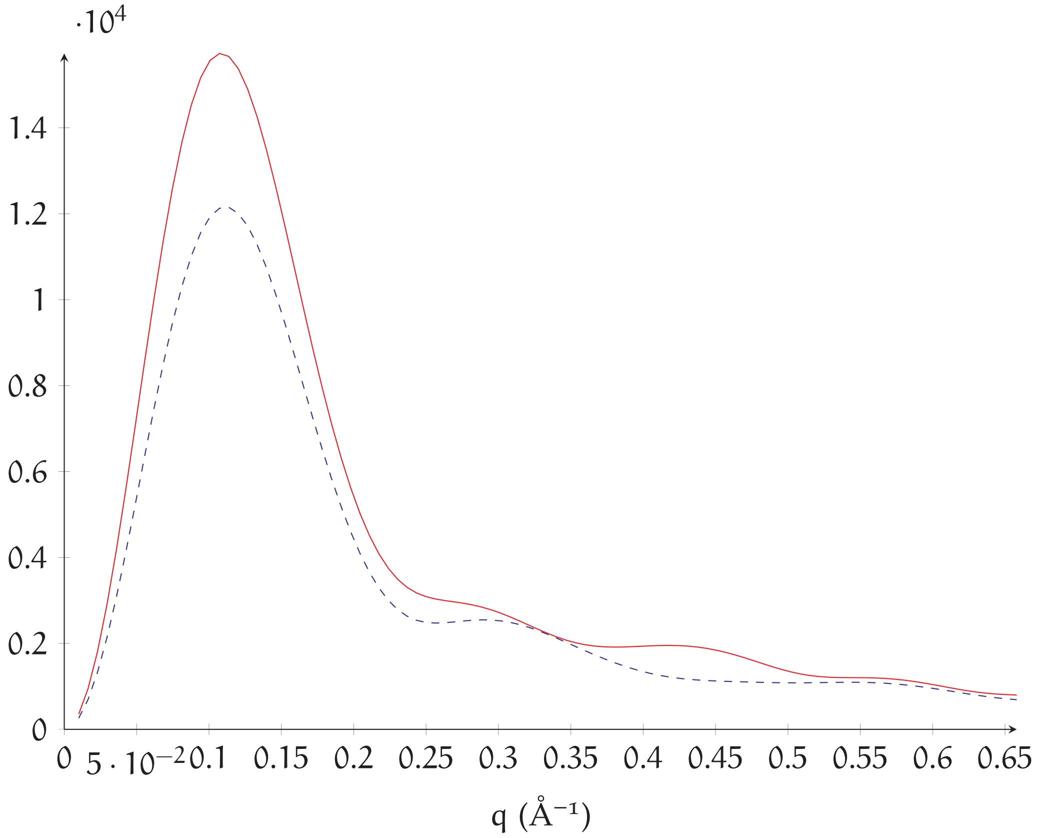

Figure 3: Lysozyme standard deviation from first (blue dashes) and second MD simulation (red solid line)
