# Supplementary material for: SAS profile correlations reveal SAS hierarchical nature and information content
Source: PLoS One. 2017 May 11;12(5):e0177309. doi: 10.1371/journal.pone.0177309 (PMC5426712; doi:10.1371/journal.pone.0177309)
Supplement: S5 Text — Similar analysis on the ensemble of the p15PAF IDP. (PDF) [file pone.0177309.s005.pdf]

S5 Text: p15PAF

Yannick G. Spill and Michael Nilges

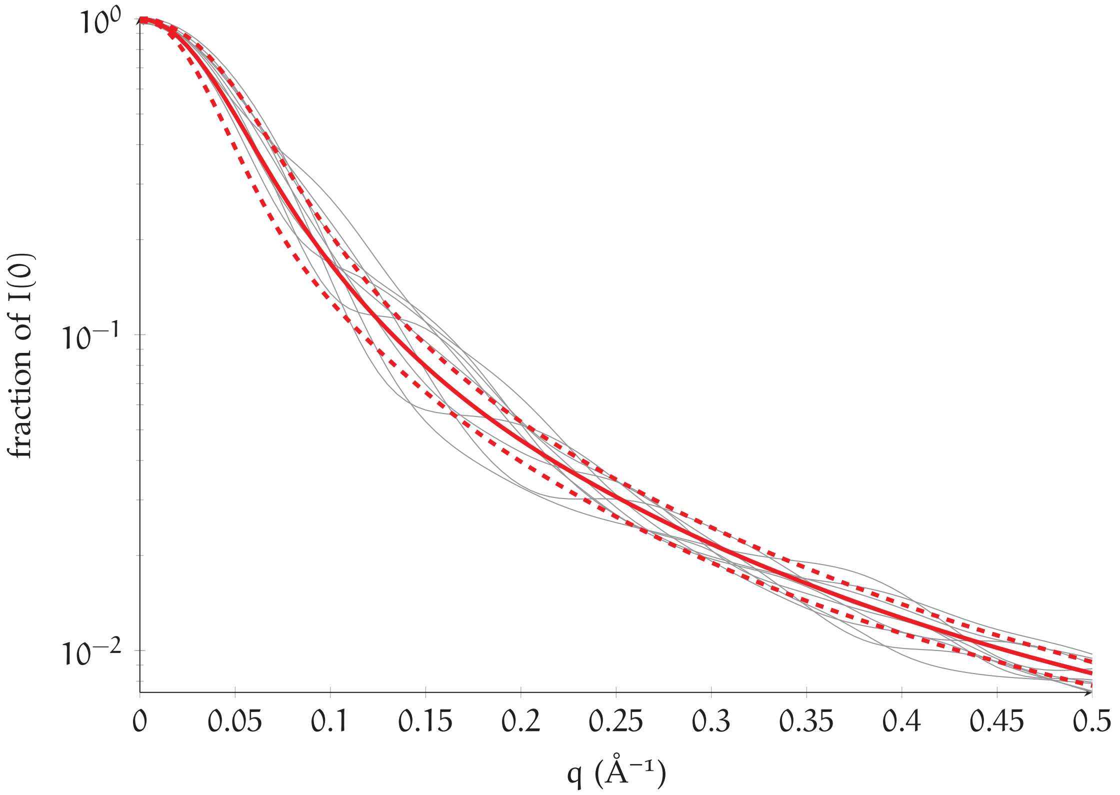

Figure 1: Ensemble average SAS profile (solid red), 68% (1σ) confidence interval (dashed red) and 9 example SAS profiles (grey) of p15PAF protein ensemble[1] (PED code PED6AAA). The deposited ensemble contains 4939 structures. Individual SAXS profiles were calculated using FoXS with $c1 = 1$ and $c2 = 0.68$.

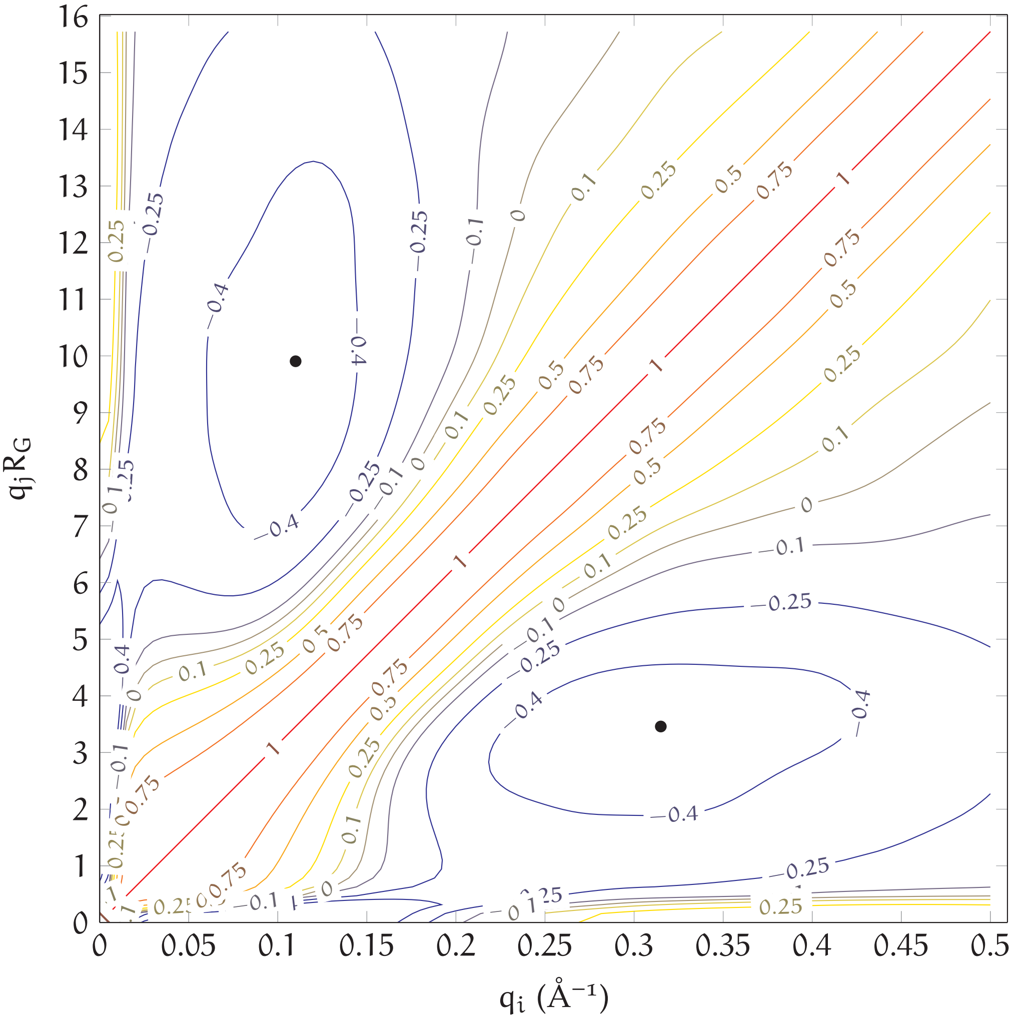

Figure 2: p15PAF SAS profile numerical correlations using ensemble deposited on PED (code PED6AAA, see text).[2] $R_G = 31.4$ Å. Smallest correlation is -0.47 and is indicated by a blue dot. The deposited ensemble contains 4939 structures. Individual SAXS profiles were calculated using FoXS with $c1 = 1$ and $c2 = 0.68$.

References

De Biasio, A, A Ibañez de Opakua, T N Cordeiro, M Villate, N Merino, N Sibille, M Lelli, T Diercks, P Bernadó, and F J Blanco (2014). "p15PAF is an intrinsically disordered protein with nonrandom structural preferences at sites of interaction with other proteins". In: *Biophys. J.* 106.4, pp. 865–874. DOI: 10.1016/j.bpj.2013.12.046.
